# Mutation of Residue βF71 of *Escherichia coli* Penicillin Acylase Results in Enhanced Enantioselectivity and Improved Catalytic Properties

**Published:** 2009-10

**Authors:** I.V. Shapovalova, W.B.L. Alkema, O.V. Jamskova, E. de Vries, D.T. Guranda, D.B. Janssen, D.B. Švedas

**Affiliations:** 1 Belozersky Institute of Physicochemical Biology and Faculty of Bioengineering and Bioinformatics, Lomonosov Moscow State University, Russia;; 2 Department of Biochemistry, Groningen Biomolecular Sciences and Biotechnology Institute, University of Groningen, Netherlands

## Abstract

Residue phenylalanine 71 of the β-chain of penicillin acylase from *E. coli* is involved in substrate binding and chiral discrimination of its enantiomers. Different amino acid residues have been introduced at position βF71, and the mutants were studied with respect to their enantioselectivity and substrate specificity. Some mutants demonstrated remarkably improved catalytic activity. Moreover, mutation of βF71 residue allowed to enhance penicillin acylase enantioselectivity. The catalytic activity to the specific substrates was improved up to 36 times, most notably for K, R, and L mutants. Increased activity to a D-phenylglycine derivative - a valuable specificity improvement for biocatalytic synthesis of new penicillins and cephalosporins - was shown for βF71R and βF71L mutants. The synthetic capacity of penicillin acylase with 6-aminopenicillanic acid as an external nucleophile was especially sensitive to mutation of the β71 residue in contrast to the synthesis with 7-aminodeacetoxycephalosporanic acid.

## INTRODUCTION

Penicillin acylase (PA) from *Escherichia coli* is probably the most studied enzyme among the group of Ntn-hydrolases. The enzymes of this superfamily are characterized by their unique catalytic mechanism in which the N-terminal amino acid residue (serine, cysteine or threonine) acts as a nucleophile to form an acylenzyme intermediate. PAs, as well as other Ntn-hydrolases, are activated by processing of a precursor protein to form the mature enzyme that has an αββα-fold topology of helices and strands around the active site [1]. Despite the high interest in the structure and biosynthesis of PAs [2-13], as well as in the new synthetic applications [14-20], little work has been done on the substrate specificity and especially the enantioselectivity of these enzymes [21, 22]. The reason for this is that the substrate specificity studies with phenylacetylated compounds are seriously complicated by a very strong competitive inhibition by the reaction product phenylacetic acid [23, 24]. PAs seem to possess a much wider substrate range than previously assumed (especially concerning the leaving group), and acylases of different origins may have quite different catalytic activities and enantioselectivities [24]. The substrate specificity and catalytic properties of PAs are of very high practical interest as this family of enzymes plays a decisive role in the biocatalytic preparation of semisynthetic β-lactam antibiotics [25].

A breakthrough in the elucidation of the catalytic mechanism of PA from *E. coli* was obtained when the X-ray crystallographic structure of the native enzyme [2, 10] and an enzyme-substrate complex was solved [12, 13]. This provided the first information about the residues involved in binding of the leaving group of the substrate. Afterwards, molecular modeling has helped to reveal the intimate details of the substrate binding in the penicillin acylase active center, especially concerning the binding pattern of the leaving group [26]. Additional contacts with residues βG385, βS386, and βN388 have been found, which were missing in X-ray structures. Based on structural information and molecular modeling, the key amino acid residues that control interactions between the enzyme and substrate have been identified. Such insight is of crucial importance for the rational redesign of basic biocatalytic properties, including substrate specificity, enantioselectivity, and the catalytic activity. Amino acid residue F71 of the β-chain of PA was demonstrated to be one of the principal amino acid residues interacting with the substrate’s leaving group. Such a residue is an attractive target for mutagenesis aimed at modifying the catalytic performance of PA for the biocatalytic modification of β-lactam antibiotics, chiral resolution of amino compounds, etc. In this paper, we report our results on the modification of the major catalytic properties of PA from *E. coli*: enhancement of the enantioselectivity and improvement of the catalytic activity. These changes were obtained by mutating a single amino acid residue (βF71) in the enzyme’s active center.

## MATERIALS | METHODS

Mutagenesis of residue βF71 was achieved using three PCR reactions. The first reaction contained the BST_FW_ primer, 5’-CAGGGAAGAACCGGGAAACTATTG-3’, as the forward primer and the F71_RV_ reverse primer carrying the mutation in the codon for βF71 at the underlined position, 5’-AAAAATATCGACATCGTCGCCCCAACCTGCCGT-3’. The second reaction contained F71_FW_ 5’-GGCGACGATGTCGATATTTTT-3’ as the forward primer and NHE_RV_ 5’-CACTCCTGCCAATTTTTGGCCTTC-3’ as the reverse primer. Products from both reactions were isolated from gel and used together in a third reaction without wild-type template, to which also the primers NHE_RV_ and BST_FW_ were added. The resulting products, carrying the mutations on position βF71, were cut with BstX1 and NheI and ligated into pEC, which was digested with the same enzymes. Ligation mixtures were used to transform competent *E. coli* cells as described [27]. PA genes from the resulting transformants were sequenced to confirm the mutations and the absence of second-site mutations that might have been introduced by PCR. Expression and purification of mutants, as well as WT PA, was done as described [12].

The concentration of active PA was determined by titration with phenylmethylsulfonylfluoride as described earlier [28]. Kinetic studies were performed by the initial rate analysis of corresponding reactions according to [24, 30]. Enantioselectivity (E) of the WT PA and its mutants was characterized as a ratio of the second order rate constants determined for the hydrolysis of the individual enantiomers E=(k_cat_/K_m_)^L^/(k_cat_/K_m_)^D^ as described earlier [21, 24].

Molecular dynamics simulations based on the available X-ray structure 1H2G of βF71 [29] were done with the Gromacs package (www.gromacs.org) as described earlier [26]. Molecular docking was performed using Lead-Finder software from MolTech Ltd., Russia (www.moltech.ru).

## RESULTS AND DISCUSSION

**Fig. 1.**
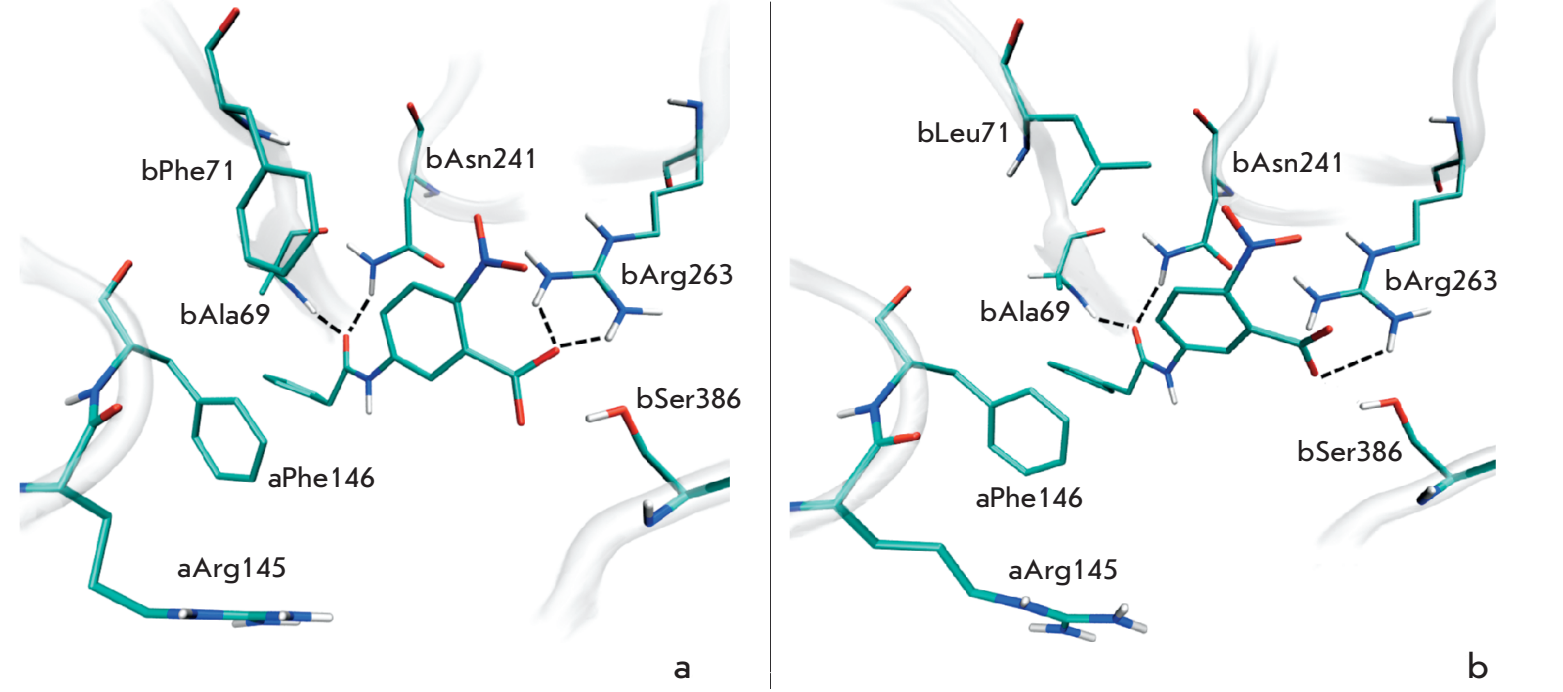
Molecular modeling of enzyme-substrate complexes for wild-type penicillin acylase (a) and its βF71L mutant (b) with N-(3-carboxy-4-nitrophenyl)phenylacetamide. Interactions of the substrate with oxyanion hole residues (βA69 and βN241) and residue βR263 are indicated by dotted lines

**Fig. 2.**
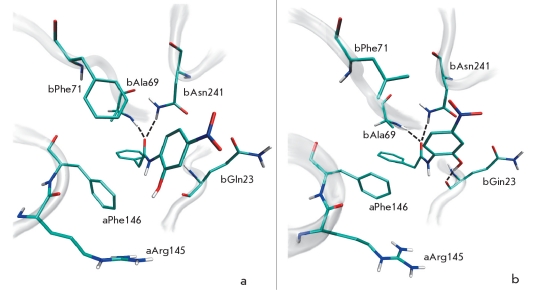
Molecular modeling of enzyme-substrate complexes for wild-type penicillin acylase (a) and its ?F71L mutant (b) with N-(2-hydroxy-4-nitrophenyl)phenylacetamide. Interactions of the substrate with oxyanion hole residues (?A69 and ?N241) and residue ?Q23 are indicated by dotted lines

Amino acid residue F71 of the β-chain slightly moves upon binding of penicillin G in the active centre of PA and stays in close proximity to the β-lactam group, displaying van der Waals interactions [12]. Molecular modeling shows that residue βF71 plays an important role in the binding of other substrates, for example N-(3-carboxy-4-nitrophenyl)phenylacetamide (Fig.1a) and N-(2-hydroxy-4-nitrophenyl)phenylacetamide (Fig.2a). Moreover, mutation of this amino acid residue (Figs.1b and 2b) enhances the substrate’s interaction with the oxyanion hole and its orientation. It therefore was expected that mutation of residue β71 might influence the catalytic activity of PA and change enantioselectivity, as well as enzyme specificity to the leaving group. Consequently, βF71 mutants containing different functionalities in the side chain were prepared and studied with respect to their enantioselectivity and catalytic properties towards substrates of different chemical structures. All mutants were catalytically active, demonstrating that mutation of the βF71 residue did not influence the processing and correct folding of the mutant enzymes. Phenylmethylsulfonylfluoride, the well known irreversible inhibitor and active site titration reagent for penicillin acylases [24, 28], was used to determine the active site concentration of the mutant enzymes. Active site titration of WT PA and each mutant allowed to determine the absolute catalytic activity and accurately compare their catalytic properties. Kinetic studies included measuring the catalytic activity in the hydrolysis of phenylacetic acid derivatives with different, but structurally related leaving groups and α-amino substituent in the acyl group. The enantioselectivity of the mutants in the hydrolysis of N-phenylacetylated amino acids was used as a quantitative measure of chiral discrimination in the leaving group binding subsite. The most remarkable changes induced by the mutation of the single βF71 residue are discussed below.

## CATALYTIC EFFICIENCY OF MUTANTS

**Fig. 3.**
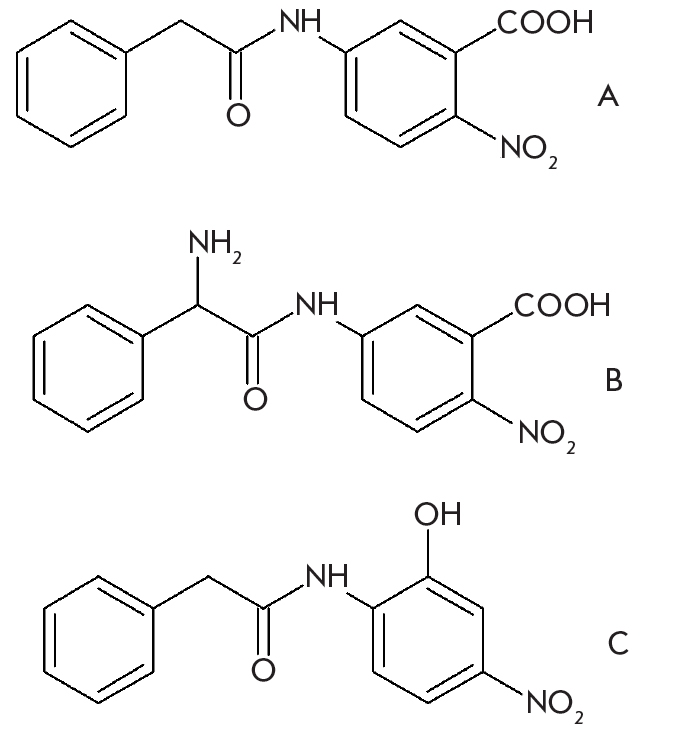
Structure of penicillin acylase substrates used to test catalytic activity of wild-type enzyme and its mutants

**Fig. 4.**
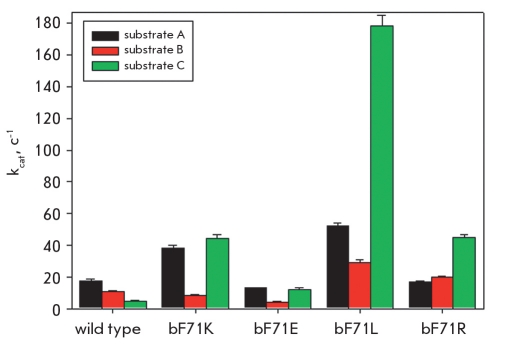
Catalytic activity of wild-type penicillin acylase and its βF71 mutants expressed as the value of the catalytic constants of the enzymatic hydrolysis of substrates presented in Fig.3

To compare the catalytic efficiency of wild-type penicillin acylase and its mutants, we measured the kinetics of the hydrolysis of chromogenic phenylacetic acid and D-phenylglycine derivatives (Fig.3). It can be seen that PA catalytic activity has changed considerably by mutating residue βF71. As expected, mutation influences the specificity to the leaving group (Fig.4). Most tested mutants demonstrated improved catalytic properties, but that was not the case with all tested substrates. The K, R, and L mutants showed a very large increase in catalytic activity only with some selected substrates, whereas the catalytic properties of the E mutants were improved to a lesser extent. βF71E mutant also had a decreased affinity to the substrates studied. Out of all tested compounds, the highest k_cat_ was found for the βF71L mutant with an improvement of more than 36 times compared to the WT PA.

In addition to the improved catalytic activity, the K, R, and L mutants also had substantially increased affinity to different substrates (data not shown), demonstrating that in general the phenylalanine at position 71 is not an optimal amino acid residue, neither for the catalytic activity nor for the affinity of PA to its substrates. It was observed that mutation of βF71 changes the properties of the acyl group binding subsite and improves PA specificity to D-phenylglycine derivatives, which are key acyl donors in enzymatic synthesis of the most important semisynthetic penicillins and cephalosporins, such as ampicillin and cephalexin. The search for new PAs that are more specific to D-phenylglycine derivatives has a long-lasting history [31-33], and there was no expectation that the specificity of PA from *E. coli* can be improved for this group of compounds. However, the βF71L mutant had increased specificity (k_cat_/K_m_ value improved by a factor of 4.4) due to the increased catalytic activity and the improved affinity to the substrate. In fact, the nature of the "optimal" residue on position β71 depends on the structure of the substrate converted. Our results clearly indicate that mutation of residue βF71 may be used to design modified PAs with improved catalytic performance.

## NUCLEOPHILE REACTIVITY STUDIES

The most straightforward quantitative way of studying the effect of mutations on the binding of β-lactam nuclei in the course of penicillin acylase-catalyzed synthesis of new β-lactam antibiotics would be to measure the corresponding binding constants. However, because of the complex kinetics of PA-catalyzed acyl transfer reactions [34] there is no proper method to determine the nucleophile binding constant to that particular subsite of the enzyme. Theoretical analysis has shown that an adequate way of characterizing the interaction of the nucleophile with the enzyme is to measure the ratio of initial synthesis and hydrolysis rates, the (S/H)_ini_ ratio, as a function of the nucleophile concentration [35]. Kinetic experiments were, therefore, performed with two different side chain donors at a fixed concentration of the β-lactam antibiotic nucleus (6-APA or 7-ADCA). The results show that the nucleophile reactivity of 6-APA is much more sensitive to the amino acid located at position β71 compared to that of 7-ADCA (Table 1). Obviously, mutation of βF71, especially substitution of the phenylalanine for a charged amino acid residue, leads to a substantial change of the β-lactam binding, and, as a result, the specificity of the leaving group binding subsite is modified.

**Table 1:** Initial synthesis/hydrolysis rate ratios (S/H)_ini_ for the ampicillin, amoxicillin and cephalexin synthesis catalyzed by wild-type penicillin acylase and its βF71 mutants using D-phenylglycine amide (D-PGA) and D-p-hydroxyphenylglycine amide (D-HPGA) as acyl donors. Experimental conditions: pH 7.0, 25°C, 0.05 M phosphate; D-PGA and D-HPGA concentration 0.015 M, 6-APA and 7-ADCA – 0.025 M.

Enzyme	D-PGA/7-ADCA	D-PGA/6-APA	D-HPGA/6-APA
wild type	9 ± 2	1.9 ± 0.1	1.2 ± 0.04
bF71Y	7.5 ± 0.4	0.74 ± 0.01	0.61 ± 0.03
bF71L	4.9 ± 0.4	0.94 ± 0.04	0.86 ± 0.03
bF71W	2.9 ± 0.2	0.77 ± 0.05	0.62 ± 0.03
bF71R	1.8 ± 0.03	0.76 ± 0.01	0.45 ± 0.01
bF71K	1.2 ± 0.01	0.51 ± 0.01	0.46 ± 0.02

## ENANTIOSELECTIVITY

**Fig. 5.**
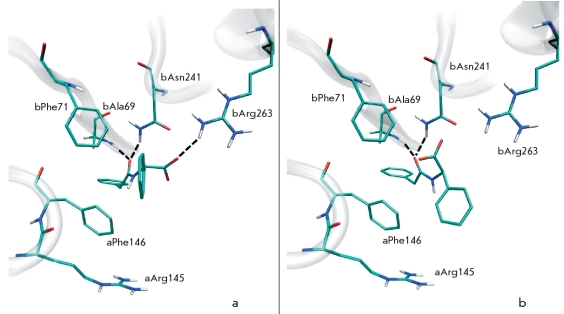
Molecular modeling of enzyme-substrate complexes for wild-type penicillin acylase with N-phenylacetyl-L-phenylglycine (a) and N-phenylacetyl-D-phenylglycine (b). Interactions of the L-form and the D-form of the substrate with oxyanion hole residues (βA69 and βN241) are indicated by dotted lines. Interaction of the substrate with residue βR263 is observed only with the L-form of the substrate

**Fig. 6.**
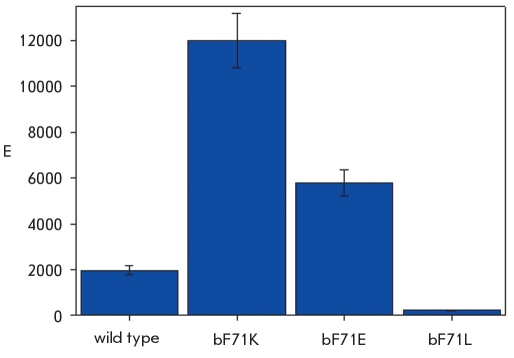
Enantioselectivity (E) of penicillin acylase mutants in the hydrolysis of N-phenylacetyl-phenylglycine, expressed as the ratio (*k*_cat_/K_m_)_L_ / (*k*_cat_/K_m_)_D_

Since residue βF71 interacts with the leaving group of the substrate, mutating it should influence PA reactivity towards the stereochemical isomers of chiral substrates. Therefore, the enzyme’s enantioselectivity value E (a ratio of k_cat_/K_m_ values for conversion of L- and D-enantiomers of the substrate) in the hydrolysis of N-phenylacetylated amino acids was chosen as a quantitative parameter to describe the effect of mutations. Although the WT PA exhibits already a very good chiral discrimination of N-phenylacetyl-phenylglycine enantiomers due to the orientation of the L-form of substrate in the active site by βR263 residue (Fig.5), it was still possible to improve it (Fig.6). The mutant that was superior in its catalytic activity, βF71L, displays a markedly decreased enantiopreference by almost one order of magnitude, while the βF71E and βF71K mutants reveal a three and six-fold increase, respectively. The positive effect of the mutation (*i.e.* the higher enantioselectivity) originated in the decreased affinity for the slow-reacting D-enantiomer, while negative effects are mainly caused by the altered reactivity of both enantiomers (data not shown). Enantioselectivity, as well as catalytic activity studies, shows that PA catalytic properties can be finely tuned by mutating the βF71 residue for the specific substrate of interest.

## CONCLUSIONS

Residue βF71 of PA from *E. coli* strongly influences the catalytic activity and chiral discrimination of the substrate’s enantiomers. Depending on the structure of the substrate, βF71 mutations may deteriorate or improve the catalytic rate, enantioselectivity, or affinity. This indicates that a universal improvement of all major catalytic properties for biocatalytic application is hardly possible, and that the enzyme should be fine-tuned for its specific substrate of interest. The availability of structure/function insight concerning specific residues and molecular modeling can facilitate the design of a toolbox of dedicated PAs for different applications.

## Acknowledgements

This work was supported by the Russian Federal Agency for Science and Innovation (federal contract 02.527.11.0001), the Seventh Framework Programme (FP7) of the European Commission and the Dutch Ministry of Economics.

## References

[1] Oinonen C., Rouvinen J.  (2000). Protein Sci..

[2] Duggleby H.J., Tolley S.P., Hill C.P., Dodson E.J., Dodson G., Moody P.C.E.  (1995). Nature.

[3] Brannigan J.A., Dodson G., Duggleby H.J., Moody P.C., Smith J.L., Tomchik D. R., Murzin A.G.  (1995). Nature.

[4] Isupov M.N., Obmolova G., Butterworth S., Badet-Denisot M.A., Badet B., Polikarpov I., Littlechild J.A., Teplyakov A.  (1996). Structure.

[5] Groll M., Ditzel L., Lowe J., Stock D., Bochtler M., Bartunik H.D., Huber R. (1997). Nature.

[6] Guo H.C., Xu Q., Buckley D., Guan C.  (1998). J. Biol. Chem..

[7] McDonough M.A., Klei H.E., Kelly J.A.  (1999). Protein Sci..

[8] Suresh C.G., Pundle A.V., Rao K.N., SivaRaman H., Brannigan J.A., McVey C.E., Verma C.S., Dauter Z., Dodson E.J., Dodson G.G. (1999). Nature Struct. Biol..

[9] Schumacher G., Sizmann D., Haug H., Buckel P., Bock A.  (1986). Nucleic Acid Res..

[10] Done S.H., Brannigan J.A., Moody P.C.E., Hubbard R.E.  (1998). J. Mol. Biol..

[11] Hewitt L., Kasche V., Lummer K., Lewis R.J., Murshudov G. N., Verma C. S., Dodson G.G., Wilson K.S.  (2000). J. Mol. Biol..

[12] Alkema W.B.L., Hensgens C.M.H., Kroezinga E.H., De Vries E., Floris R., Van der Laan J.-M., Dijkstra B.W., Janssen D.B.  (2000). Protein Eng..

[13] McVey C.E., Walsh M.A., Dodson G.G., Wilson K.S., Brannigan J.A.  (2001). J. Mol. Biol..

[14] Baldaro E., D’Arrigo P., Pedrocchi-Fantoni G., Rosell C.M., Servi S., Tagliani A., Terreni M. (1993). Tetrahedron: Asymmetry.

[15] Waldmann H., Sebastian D.  (1994). Chem. Rev..

[16] Soloshonok V.A., Soloshonok I.V., Kukhar V.P., Svedas V.K.  (1998). J. Org. Chem..

[17] Topgi R.S., Ng J.S., Landis B., Wang P., Behling J.R.  (1999). Bioorg. Med. Chem..

[18] Basso A., De Martin L., Ebert C., Gardossi L., Linda P. (2001). J. Mol. Cat. B: Enzymatic.

[19] Guranda D.T., Van Langen L.M., Van Rantwijk F., Sheldon R.A., Svedas V.K.  (2001). Tetrahedron: Asymmetry.

[20] Guranda D. T., Khimiuk A.I., Van Langen L.M., Van Rantwijk F., Sheldon R. A., Svedas V.K.  (2004). Tetrahedron: Asymmetry.

[21] Svedas V.K., Savchenko M.V., Beltser A.I., Guranda D.F.  (1996). Acad. Sci..

[22] Galunsky B., Lummer K., Kasche V.  (2000). Monatsh. Chem..

[23] Berezin I.V., Klyosov A.A., Nys P.S., Savitskaya E.M., Svedas V.K.  (1974). Antibiotiki.

[24] Svedas V.K., Guranda D.T., Van Langen L.M., Van Rantwijk F., Sheldon R.A.  (1997). FEBS Lett..

[25] Bruggink A., Roos E.C., De Vroom E.  (1998). Org. Process Res. Dev..

[26] Chilov G.G., Stroganov O.V., Svedas V.K.  (2008). Biochemistry.

[27] Sambrook J., Fritsch E. F., Maniatis T.  (1989). Molecular cloning a laboratory manual 2nd.

[28] Svedas V.K., Margolin A.L., Sherstyuk S.F., Klyosov A.A., Berezin I.V.  (1977). Bioorg. Khim..

[29] Morillas M., McVey C.E., Brannigan J.A., Ladurner A.G., Forney L.G., Virden L.  (2003). Biochem.J..

[30] Youshko M.I., Shamolina T.A., Guranda D.F., Sinev A.V., Svedas V.K.  (1998). Biochemistry.

[31] Takahashi T., Yamazaki Y., Kato K., Isona M. (1972). J. Am. Chem. Soc..

[32] Blinkovsky A.M., Markaryan A.N.  (1993). Enzyme Microb. Technol..

[33] Polderman-Tijmes J.J., Jekel P.A., van Merode A., Floris T.A.G., van der Laan J.-M., Sonke T., Janssen D.B.  (2002). Appl. Environ. Microbiol.

[34] Youshko M.I., Svedas V.K.  (2000). Biochemistry.

[35] Youshko M.I., Chilov G.G., Shcherbakova T.A., Svedas V.K.  (2002). Biochim. Biophys. Acta: Proteins Amp; Proteomics.

